# The times they are a changing

**DOI:** 10.4102/sajid.v37i2.376

**Published:** 2022-02-14

**Authors:** Lesley M. Devenish

**Affiliations:** 1 Department of Clinical Microbiology and Infectious Diseases, Faculty of Health Sciences, University of the Witwatersrand, Johannesburg, South Africa

My implantation into infection prevention and control (IPC) occurred quite by chance. I was invited to give a talk on measles at a hospital and stayed to set up the Infection Control Department (as it was known then) in that hospital. After a quick 3-day introductory course with the legendary Joan Pearse, I was on my way into an exciting, ever-changing field that continues to expand each year and has taken me all over the world.

In those years, our most notorious ‘bug’ was methicillin-resistant *Staphylococcus aureus* (MRSA). HIV was still a teenager, and the concomitant epidemic of HIV/tuberculosis was yet to raise its ugly head in South Africa. The rise of infections with Gram-negative bacteria and *Clostridioides difficile* (these name changes!) was also yet to come.

This was followed by the ‘millennium bug’ years and with it, keeping up with myriad changes in microbiology. I went from a growing realisation of the importance of hand hygiene to the entrenchment of this most important of measures in my psyche. The World Health Organization’s ground-breaking launch of the first Global Patient Safety Challenge introduced the ‘My 5 Moments For Hand Hygiene’ in 2009. Yet, I find my IPC students today still struggling to remember them all. It is sad that compliance with hand hygiene remains a behavioural challenge. Along with many others, I am sure, my ongoing wish is for genetically modified visible microbes on hands and surfaces.

The coronavirus disease 2019 (COVID-19) pandemic has taught us to make hand and surface hygiene relevant to the issue we face now, which is equally as much about transmission through the air. The debate about droplet versus airborne transmission has fascinated me from the time I was introduced to these concepts – where does the one stop and the other begin? I am grateful for the engineers who waded into the debate and explained that ‘airborne’ transmission is, in fact, a continuum, and that context is more important (proximity, ventilation and space). The pandemic also emphasised the importance of IPC interventions in the community; that using gloves badly is worse than not using gloves at all, and that it is really annoying to have to use eye protection.

Grasping the idea that our human gut microbiome is essential to our health was an eye-opening concept to IPC practitioners, who, let’s face it, are a bunch of germophobes. The fairly recent research on the role of sinks in the transmission of Gram-negative organisms was scary – we had long promoted having more sinks to increase compliance with hand hygiene, only to have another pendulum swing – to advocating for the shielding or indeed the removal of sinks in intensive care units! We went from viewing the sink as our best friend in IPC to acknowledging it as a waste disposal point!

The Gram-negative bacteria in Enterobacteriaceae (with a name change to Enterobacterales) became a significant challenge for IPC nurses. We learned the value of screening in our arsenal! Antibiotic resistance, in general, has now become a battle of almost intergalactic scale, with carbapenem-resistant Enterobacterales being the current Death Star. I say current because we know for sure that there will be new challenges.

We have come far, but how far we have yet to go!

I believe that the biggest failing as IPC nurses is not talking to doctors and administrators more (and, by extension, politicians). It is only by influencing these groups that we can achieve a better connection with and funding of IPC in a world of spiralling healthcare costs. Also, IPC training should be integral to medical, nursing and allied health sciences training.

As we move towards a ‘living with COVID-19’ approach (how everyone hated the phrase ‘the new normal’), we need to look at IPC practitioners becoming more involved in antimicrobial stewardship, embracing digital systems (so hard for nurses who do not work with computers every day), embedding the ‘bundles of care’ for the four major acute healthcare-associated infection (central line-associated bloodstream infection [CLABSI], catheter-associated urinary tract infection [CAUTI], ventilator-associated pneumonia [VAP], surgical site infection [SSI]), which, sadly to say, are not yet implemented in all hospitals, connecting deeply with the revolution of quality improvement in health care, and striving to become the kind of IPC practitioner to whose advice hospital management actually listens and acts upon.

My advice to new Infection Preventionists is to read, read, read, and then read some more. Sign up for the free IPC-related websites; attend as many of the free webinars as you can; and participate in journal club meetings and attend IPC training days, seminars and conferences, when those days come back again. Become good friends with your ever-approachable and helpful microbiologists. Moreover, get involved and foster positive, professional relationships with the healthcare team. Promote vaccination! Work on becoming more computer literate, and install those helpful apps. Always lead with enthusiasm, energy and insight and let your colleagues see your passion and commitment.

And always, always, set the example at every opportunity (get rid of those painted nails forevermore)! Infection Prevention and Control is, after all, a behavioural science.

